# Bilateral thigh compartment syndrome following intraoperative pelvic binder reduction of open pelvic fracture: a case report and review of literature

**DOI:** 10.1007/s00590-024-04090-7

**Published:** 2024-09-06

**Authors:** Phillip Chung, Ian G. Hasegawa, Andrew M. Duong, Soroush Shabani, Joseph T. Patterson

**Affiliations:** 1 Department of Orthopaedic Surgery, Keck School of Medicine of the University of Southern California, 1520 San Pablo Street, Suite 2000, Los Angeles, CA 90033-5322 USA; 2 Queen’s University Medical Group, 1301 Punchbowl Street, Honolulu, HI 96813 USA

## Abstract

**Case:**

A 22-year-old man with a type IIIA open AO/OTA 61C2.2b pelvis fracture and hypotension received exploratory laparotomy, temporary open ligation of the bilateral internal iliac arteries, and retroperitoneal packing. After prompt fracture debridement, a pelvic binder was positioned over the thighs as a reduction aid and maintained for six hours during pelvis open reduction internal fixation. Bilateral anterior thigh compartment syndrome was diagnosed three hours after packing and binder removal.

**Conclusion:**

Prolonged application of a pelvic binder to the thighs as an intraoperative reduction tool, shortly after temporary internal iliac artery ligation, may be associated with reperfusion injury and thigh compartment syndrome.

## Introduction

Pelvic fractures resulting from high-energy trauma increased in incidence from 27 to 34 per 100,000 capita between 1990 and 2007 [1]. Open pelvic fractures, with a 14% mortality rate and a 50% complication rate, are particularly devastating injuries [2]. While mortality from high-energy pelvic fractures has decreased over time to 5%, massive noncompressible hemorrhage remains the leading cause of death among patients presenting with hypotension and pelvic fracture [3, 4].

Early aggressive resuscitation with application of a pelvic circumferential compression device (PCCD) such as a pelvic binder or sheet and clamps reduces mortality by mitigating the hemorrhage associated with volume-expanding pelvic ring injuries [5]. Typically used in conjunction with resuscitation, PCCDs serve as an adjunct to angiographic embolization and/or pelvic packing [6]. By applying an internal rotation torque to each hemipelvis, a PCCD is also a useful reduction aid for percutaneous and open management of “open book” pelvic ring disruptions [6]. However, prolonged application of a PCCD carries potential complications including soft tissue necrosis, nerve palsy, and bladder incarceration [7–10].

Acute compartment syndrome (ACS) is defined as increased pressure within a closed osteofascial compartment impairing local circulation [11]. ACS of the thigh (ACST) is rare because of the increased compliance of the thigh myofascial compartments, which also can make clinical diagnosis of ACST difficult [12, 13]. Invasive thigh compartment pressure measurements 30 mm Hg greater than diastolic blood pressure are concerning for ACST if corroborated by clinical signs and symptoms [11]. Complications can include muscle necrosis, rhabdomyolysis, nerve damage, infection, renal dysfunction, and multiorgan failure with a mortality rate as high as 47% [13]. Treatment of ACST includes immediate fasciotomy of the affected compartments, possible debridement, meticulous hemostasis, and delayed secondary closure of skin or skin grafting [11–13].

To our knowledge, ACST associated with intraoperative use of a PCCD as a reduction tool has not previously been reported.

## Case report

**Fig. 1:**
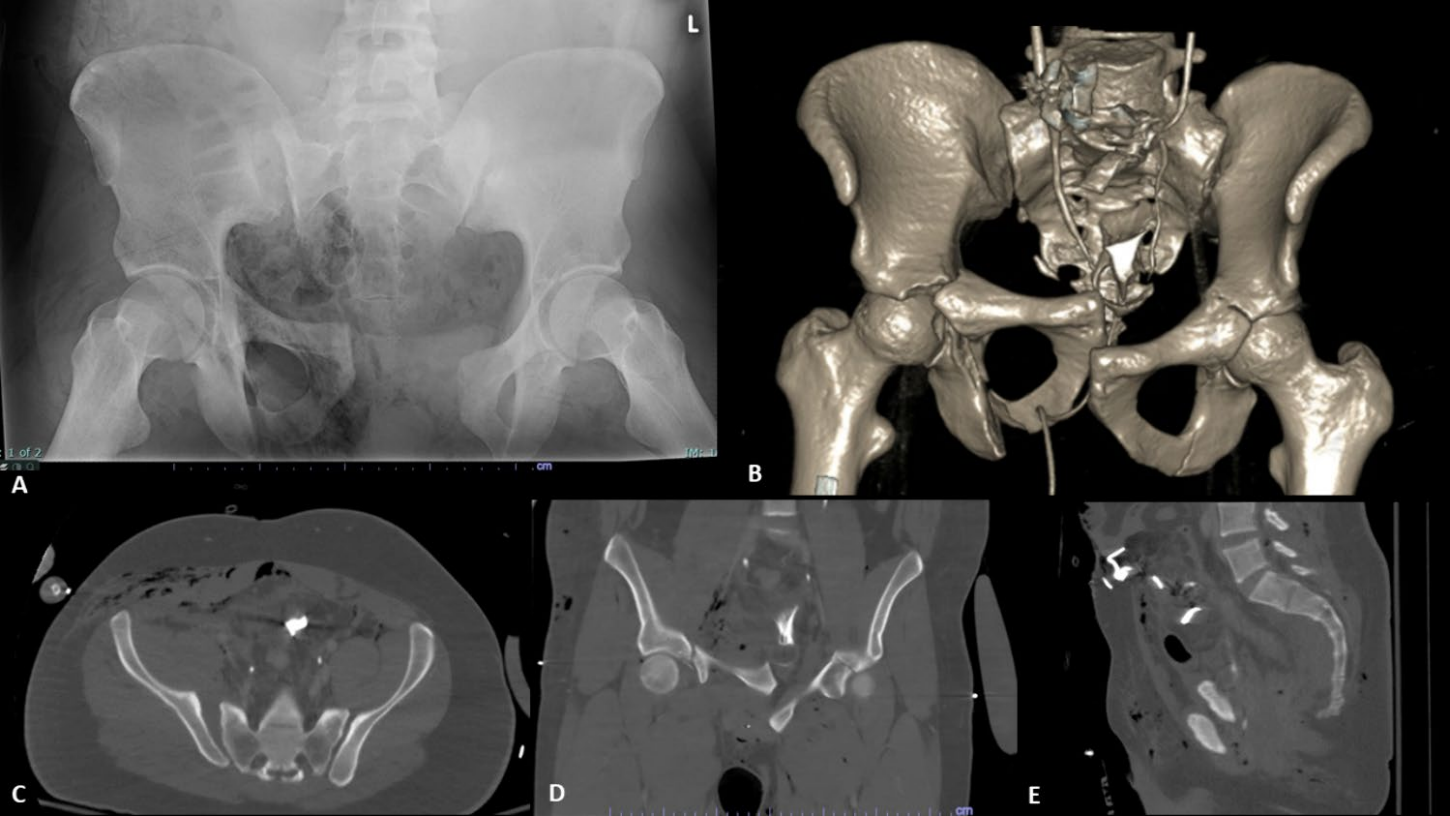
**A** Initial AP pelvis radiograph per ATLS protocol without circumferential pelvic compression. **B** 3D surface reconstruction and **C** axial, **D** coronal, **E** sagittal representative images of pelvic CT scan after application of circumferential pelvic compression

A 22-year-old man arrived at our Level 1 trauma center with a Glasgow coma scale of 12, significant facial trauma, active hemorrhage from a right inguinal laceration with open pelvic fracture, and class III hemorrhagic shock after being struck by a vehicle while operating a motorcycle. Circumferential pelvic compression was applied with a pelvic sheet. Radiographs and CT scan of the pelvis demonstrated a type IIIA open AO/OTA 61C2.2b pelvic ring disruption with complete disruption of the right sacroiliac joint, partial injury to the left sacroiliac joint, and bilateral pubic root and ischiopubic segment fractures involving the anterior wall of the acetabulum (Fig. 1). On physical examination, there was a right perineal laceration posterior to the scrotum spanning to the right inferior pubic ramus fracture and a right inguinal puncture wound with resulting right thigh hematoma.

Approximately two hours after arrival, an emergent exploratory laparotomy was performed with temporary vessel loop ligation of the bilateral internal iliac arteries and retroperitoneal packing for persistent hypotension. The patient sustained 50 cubic centimeters of blood loss during this operation in addition to one liter of blood loss in the emergency room. He received two units of packed red blood cells (pRBC) in the emergency department and required four units pRBC, one liter of crystalloid, and two units of fresh frozen plasma during the operation. In a hybrid vascular operative suite two and a half hours later, the left internal iliac artery demonstrated extravasation under fluoroscopy, suggesting active hemorrhage and necessitating GelFoam embolization. The right internal iliac artery was spared and temporary ligatures were removed three hours after initial applications. The laparotomy was later re-explored without closure of the abdomen.

**Fig. 2:**
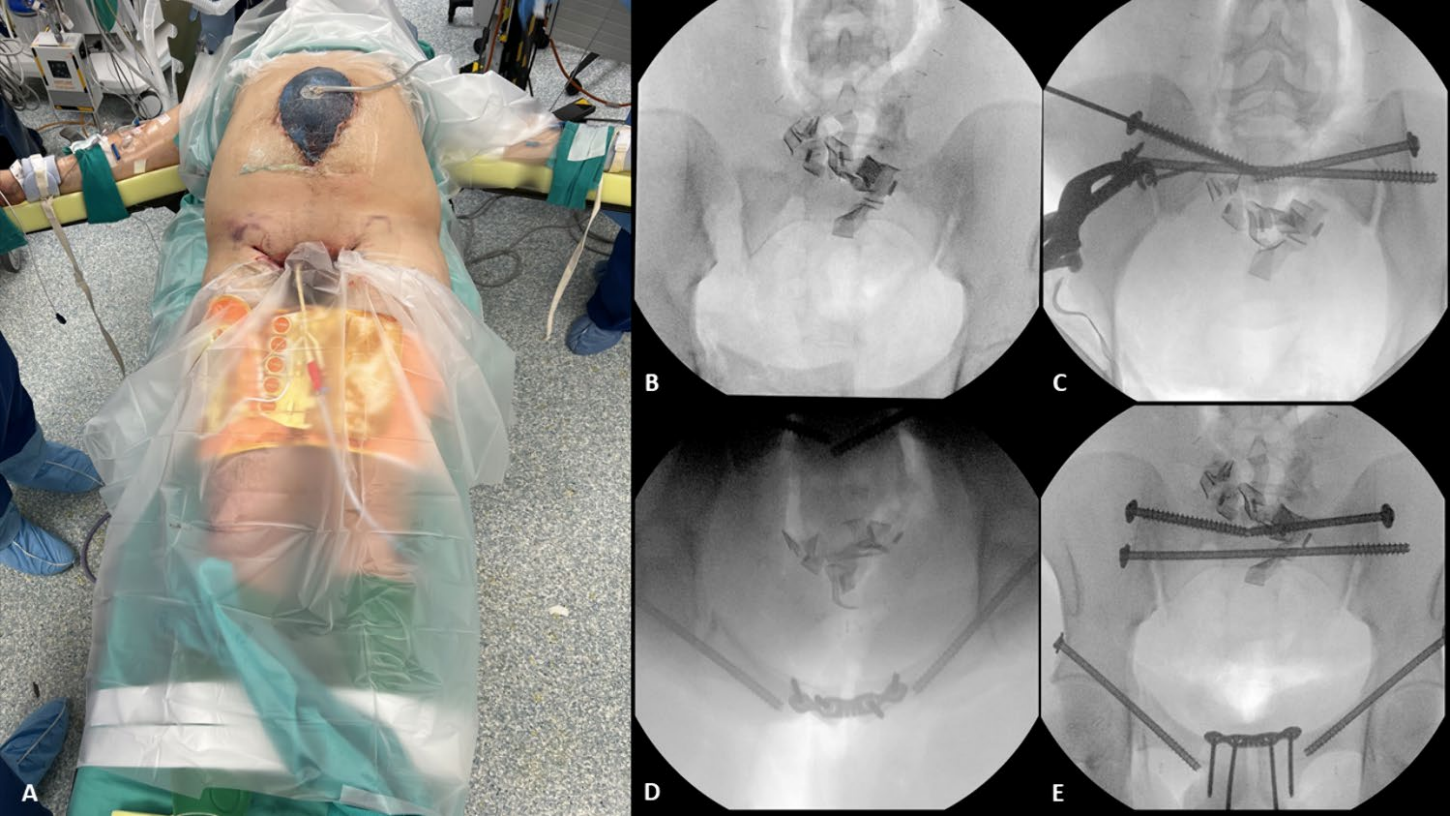
**A** Clinical photograph of pelvic binder placement prior to skin prep for skeletal stabilization of the pelvic ring injury **B**-**E** intraoperative fluoroscopy following percutaneous bilateral internal fixation of the anterior column fractures, open reduction internal fixation of the pubic symphysis and right sacroiliac joint, and percutaneous internal fixation of the bilateral posterior pelvis

Twenty-two hours after arrival, the patient underwent debridement and irrigation of the open pelvic fracture. A pelvic binder was then applied to the thighs with routine compressive force as a reduction aid via internal rotation and adduction of the thighs (Fig. 2). As shown in the fluoroscopic inlet image in Fig. 2B, closed reduction did not provide a satisfactory reduction of the right posterior sacroiliac joint despite iterative application of the pelvic binder and internal rotation of the lower extremities. After reviewing an intraoperative arterial blood gas and confirming with the anesthesia team that the patient was appropriately resuscitated and sufficiently stable for an open surgical approach, a multidisciplinary team decision was made to proceed with early appropriate care. A definitive open reduction of the right sacroiliac joint complete posterior pelvic ring injury was performed through Pfannenstiel and lateral windows using a screw-based clamp with bilateral S1 sacroiliac screws, S2 transsacral transiliac screw, bilateral antegrade anterior column screws, and symphyseal plating. The laparotomy was explored, packing removed, and the abdomen closed with no new violation of the peritoneum identified. The pelvic binder was removed six hours after application.

Three hours after binder removal, bilateral thigh swelling concerning for compartment syndrome was observed in the sedated and intubated patient. Anterior thigh compartment pressures > 40 mm Hg were measured with a needle-based monitor (Stryker, Kalamazoo, MI). Compartment pressures 10–20 mm Hg were observed in the buttock and leg compartments with palpable pedal pulses and diastolic pressure of 65 mm Hg. Bilateral thighs were tense, urine was dark brown, electrocardiogram demonstrated mild peaked T waves, and range of motion of the right knee was reduced. Laboratory studies were notable for creatine kinase > 50,000 U/L and hyperkalemia 7.4 mmol/L consistent with ACST complicated by rhabdomyolysis.

The patient was subsequently taken for emergent fasciotomies of the bilateral anterior and posterior compartments 15.5 h after open reduction of the pelvic fracture. During intraoperative examination before incision, bilateral anterior compartments were tense and bulging, and no significant swelling was visible in the posterior compartments. After fasciotomy, the right thigh demonstrated patches of congested, dark purple areas of muscle without necrosis. No debridement was performed. The left thigh after fasciotomy contained patches of nonviable quadriceps muscle unresponsive to cautery which were debrided. The majority of the left quadricep was viable. Postoperatively, continuous renal replacement therapy was initiated for the metabolic and electrolyte derangements.

The patient developed a Fournier’s gangrenous polybacterial and fungal infection of the right inguinal open fracture wound requiring multidisciplinary management including diverting colostomy, scrotal debridement, and removal of the pubic symphysis and right anterior column implants. The patient developed a ventilator-associated pneumonia with Aspergillus fumigatus and Enterobacter cloacae identified on bronchoalveolar lavage. Despite extensive treatment with broad antibiotic and antifungal agents, his traumatic facial wounds developed a surgical site infection which progressed to a necrotizing soft tissue infection involving the chest wall. The patient expired 44 days after injury due to disseminated intravascular coagulopathy in the context of acute liver failure, multiorgan dysfunction, septic shock, acute respiratory distress syndrome, and anoxic brain injury.

## Discussion

We report bilateral acute compartment syndrome of the thighs associated with intraoperative use of a pelvic binder as a reduction tool during the acute definitive fixation of an open, unstable, volume-expanding pelvis fracture following temporary bilateral internal iliac artery ligation, embolization, and retroperitoneal packing. To our knowledge, bilateral ACST has not been reported as a complication of either early definitive pelvic fracture fixation or application of a pelvic binder/sheet as a reduction tool during pelvis fracture surgery. The etiology of this complication is likely multifactorial, with potential contributions from the timing and technique of the resuscitative open and endovascular interventions, ongoing aggressive resuscitation, swelling from fluid shifts to interstitial “third space,” initial muscle damage, reperfusion injury with release of the internal iliac ligatures and removal of retroperitoneal packing, and the duration and tension of pelvic binder application.

Rapid stabilization of the pelvic ring is crucial to hemorrhage control in unstable pelvic ring injuries. The management of this patient by prompt PCCD application, resuscitation, hemorrhage control, open fracture debridement within 24 h, and early definitive fixation conforms to recommendations put forth by the Eastern and Western trauma society guidelines and a recent international survey [14–17]. Early definitive open reduction internal fixation of pelvic ring disruptions and acetabular fractures is also purported to be safe and perhaps preferable to delayed definitive treatment [17, 18].

Alternative methods for rapid stabilization of pelvic injuries include external fixation [19]. A multitude of cadaver and retrospective clinical studies have demonstrated that external fixation does not control C type, complete posterior ring injury nor provide sufficient compression to address ongoing retroperitoneal hemorrhage as seen in this patient [19–23]. In consultation with the anesthesia team, appropriate steps were taken to ensure patient stability prior to open reduction in accordance with early appropriate care.

A PCCD is also an effective reduction tool, as demonstrated by percutaneous fixation of pelvis fractures through working portals made in a PCCD [24]. Accurate placement and tightening of the PCCD provides a compressive effect which internally rotates, adducts, and medializes each hemipelvis [25–27]. A well-placed PCCD thereby can effectively reduce a B-type posterior pelvic ring injury using the intact posterior sacroiliac ligaments as a hinge. Pelvic binders, commonly applied for hours to days during resuscitation and temporary fracture stabilization, exert pressure exceeding levels associated with skin breakdown [28, 29]. A common practice is alternating pelvic binder placement between the greater trochanters and thighs for 12–24 h to reduce the risk of skin breakdown at either site [30]. Thus, pelvic binder application to the thighs as an intraoperative reduction aid for the six-hour duration in this case is within the standards of care.

Despite our adherence to current national trauma society guidelines and international standards of practice, our patient developed bilateral ACST following the intraoperative use of a pelvic binder as a reduction aid during the early definitive internal fixation of an unstable volume-expanding pelvic ring injury. We cannot determine whether the pelvic binder played a causal role in the development of ACST in this patient. ACST is a rare but described complication of pelvic fracture and has been observed in stable pelvic ring fracture in the absence of internal fixation or PCCD application [12]. A concomitant femur fracture is not necessary or sufficient for ACST; indeed, the pressure of body weight alone may be sufficient to cause ACST after trauma [13]. Reperfusion injury, a known complication in trauma cases involving temporary internal iliac vascular occlusion, is also associated with ACST [31, 32].

In this case, reperfusion injury was expected following the exploratory laparotomy and complete ligation of the bilateral iliac system with release of the ligatures in the hours prior to pelvis fracture surgery [32]. Aggressive resuscitation to address traumatic and surgical blood losses is also a known risk factor for ACST even without extremity injury [33]. It is plausible crystalloid administration during pelvis fracture fixation shortly after reperfusion of the upper thighs and buttocks by release of the internal iliac ligatures shifted fluid into the extracellular space as the osmolality of the intravascular space declined. This may have been magnified by a reperfusion inflammatory response upon release of the pelvic binder after pelvis fixation in the compressed buttocks and thighs of this muscular patient. However, ACST caused by a vascular injury is very rare and the few reported cases involve the profunda femoris vein, femoral artery dissection, or external iliac arteries [13, 34–36]. In this patient, reperfusion injury may have preferentially affected the right thigh as the right internal iliac artery was temporarily ligated, while the left was ligated and then embolized without a period of reperfusion. Despite this, ACST was observed to involve the bilateral thighs. Temporary occlusion of the internal iliac arteries may have contributed to the compartment syndrome. However, the patient’s clinical course is more consistent with alternative etiologies, and the timing of ACST diagnosis in this case correlates with binder release.

In our case review, we noticed development of muscle necrosis nine hours after initiation of the pelvic binder with application of the binder for a total of six consecutive hours. It is possible that the cause of the muscle necrosis was the initial trauma causing crush injury to the muscles. However, the initial injury was restricted primarily to the right inguinal and perineal region and no obvious deformity was noted on the left lower extremity to indicate severe injury necessary to cause muscle damage. In addition, presentation of crush injuries correlates to rhabdomyolysis [37]. In this patient, an exponential rise in creatinine kinase and potassium occurred only after prolonged binder therapy, likely indicating worsening muscle injury as a direct consequence of ACST rather than an initial presentation of acute crush injury.

 PCCDs effectively control hemorrhage and aid in reducing pelvic fractures. However, orthopedic trauma surgeons and surgical intensivists should be aware of the possible risk of ACST in the context of reperfusion following temporary bilateral internal artery ligation, thigh trauma, and volume-expanding pelvic fracture. A surgical strategy for early pelvic fracture fixation which relies on prolonged PCCD application may not be the safest approach early in the care of a patient with recent reperfusion of the internal iliac vessels.
